# Paediatric European Risperidone Studies (PERS): context, rationale, objectives, strategy, and challenges

**DOI:** 10.1007/s00787-013-0498-3

**Published:** 2013-12-15

**Authors:** Jeffrey Glennon, Diane Purper-Ouakil, Mireille Bakker, Alessandro Zuddas, Pieter Hoekstra, Ulrike Schulze, Josefina Castro-Fornieles, Paramala J. Santosh, Celso Arango, Michael Kölch, David Coghill, Itziar Flamarique, Maria J. Penzol, Mandy Wan, Macey Murray, Ian C. K. Wong, Marina Danckaerts, Olivier Bonnot, Bruno Falissard, Gabriele Masi, Jörg M. Fegert, Stefano Vicari, Sara Carucci, Ralf W. Dittmann, Jan K. Buitelaar, The PERS Consortium

**Affiliations:** 1 Department of Cognitive Neuroscience, Donders Institute for Brain, Cognition and Behavior, RUNMC Radboud University Nijmegen Medical Center, Nijmegen, The Netherlands; 2 Médecine Psychologique de l’Enfant et de l’Adolescent, CHRU Montpellier-Hôpital Saint Eloi, 80 Avenue Augustin Fliche, 34295 Montpellier Cedex 5, France; 3 INSERM Team 1, INSERM U894-Team 1, Center of Psychiatry and Neurosciences, Paris, France; 4 Department of Biomedical Sciences, Cagliari University Hospital, University of Cagliari, Cagliari, Italy; 5 Department of Psychiatry, University Medical Center Groningen, Groningen, The Netherlands; 6 Centre for Interventional Paediatric Psychopharmacology, South London and Maudsley NHS Foundation Trust, London, UK; 7 Department of Child Psychiatry, King’s College London, London, UK; 8 Child and Adolescent Psychiatry Department, Facultad de Medicina, Hospital General Universitario Gregorio Marañón, IiSGM, Universidad Complutense, CIBERSAM, Madrid, Spain; 9 Division of Neuroscience, Ninewells Hospital and Medical School, Medical Research Institute, University of Dundee, Dundee, Scotland; 10 Department of Child and Adolescent Psychiatry/Psychotherapy, University of Ulm, Ulm, Germany; 11 FCRB, Department of Child and Adolescent Psychiatry and Psychology, Neurosciences Institute, Hospital Clínic of Barcelona, SGR1119, IDIBAPS, University of Barcelona, CIBERSAM, Barcelona, Spain; 12 School of Pharmacy, University College London, University of London, London, UK; 13 Department of Practice and Policy, Centre for Paediatric Pharmacy Research, UCL School of Pharmacy, London, UK; 14 Department of Pharmacology and Pharmacy, Centre for Safe Medication Practice and Research, University of Hong Kong, Pokfulam, Hong Kong; 15 Department of Neurosciences, UPC Katholieke Universiteit Leuven, Leuven, Belgium; 16 Unité Universitaire de Psychiatrie de l’Enfant et de l’Adolescent, Nantes, France; 17 INSERM U669, Maison de Solenn, Paris, France; 18 IRCCS Fondazione Stella Maris, Pisa, Italy; 19 IRCCS Children Hospital Bambino Gesu, Rome, Italy; 20 CIMH, Central Institute of Mental Health, Mannheim, Germany; 21 Department of Psychiatry, University of Groningen, Groningen, The Netherlands

**Keywords:** Conduct disorder, Randomised clinical trials, Risperidone, Pharmacovigilance, Psychopharmacology

## Abstract

In children and adolescents with conduct disorder (CD), pharmacotherapy is considered when non-pharmacological interventions do not improve symptoms and functional impairment. Risperidone, a second-generation antipsychotic is increasingly prescribed off-label in this indication, but its efficacy and tolerability is poorly studied in CD, especially in young people with normal intelligence. The Paediatric European Risperidone Studies (PERS) include a series of trials to assess short-term efficacy, tolerability and maintenance effects of risperidone in children and adolescents with CD and normal intelligence as well as long-term tolerability in a 2-year pharmacovigilance. In addition to its core studies, secondary PERS analyses will examine moderators of drug effects. As PERS is a large-scale academic project involving a collaborative network of expert centres from different countries, it is expected that results will lead to strengthen the evidence base for the use of risperidone in CD and improve standards of care. Challenging issues faced by the PERS consortium are described to facilitate future developments in paediatric neuropsychopharmacology.

**Electronic supplementary material:**

The online version of this article (doi:10.1007/s00787-013-0498-3) contains supplementary material, which is available to authorized users.

## Introduction

The prescription of psychotropic medication is becoming an increasingly important component of comprehensive treatment programmes of child and adolescent psychiatric disorders. However, most psychotropic medications have not yet been approved for use in children or adolescents, or are only approved for a minority of indications or specific populations. One class of psychotropic medication that is increasingly being prescribed to children and adolescents is the second-generation antipsychotics (SGAs). Within this class, risperidone is the most frequently prescribed drug in children and adolescents across most countries [1–3]. In the US, the number of children receiving SGAs increased by 62 % between 2002 and 2006, especially in those with attention deficit/hyperactivity disorder and conduct disorder [4]. Data from the US National Ambulatory Medical Care Surveys between 2005 and 2009 show that disruptive behaviour disorders were the most common diagnoses in both child and adolescent visits where an antipsychotic was prescribed, counting for 63.0 and 33.7 %, respectively [5]. In the UK, the prescribing prevalence of antipsychotics for patients 7–12 years of age in general practises almost tripled between 1992 and 2005 with SGA prescribing increasing 60-fold from 1994 to 2005 [6]. In the Netherlands, prescribing prevalence of antipsychotics doubled from 1997 to 2005 with the highest prevalence being among 10- to 14-year olds, and especially among boys [7]. This increased prevalence was mainly attributable to both increased use of SGAs and a longer duration of use, which clearly increases the exposure of this young population to incompletely understood risks [8, 9].

These increased prescription rates of risperidone and other SGAs for children and adolescents, particularly for non-psychotic conditions, the off-license status of many of these prescriptions, their metabolic side effects and the inadequacy of long-term surveillance have raised concern about uncritical prescriptions [2, 10, 11]. This context also provided the impetus for the development of the Paediatric European Risperidone Studies (PERS) research programme that was awarded funding by the EU 7th framework in response to the call HEALTH-2009-4.2-1: “Adapting off-patent medicines to the specific needs of paediatric populations”. PERS focuses on the use of risperidone in children and adolescents with CD, and its key objective is to perform a series of clinical studies, underpinned by a Paediatric Investigation Plan (PIP) that will provide sufficient information for a Paediatric Use Marketing Authorisation (PUMA) to be obtained for the use of risperidone in CD in children and adolescents. A PUMA is a dedicated marketing authorisation for medicinal products indicated exclusively for use in the paediatric population. PUMAs were introduced by the European Medicines Agency to encourage the development of paediatric medicines. (http://www.ema.europa.eu/ema/index).

In this paper, we describe the rationale of the PERS studies, which include non-commercial clinical trials and an observational study on the efficacy and safety of risperidone in children and adolescents with CD and without intellectual disability.

## Conduct disorder

Conduct disorder is a clinically heterogeneous disorder defined by a pattern of repetitive and persistent antisocial behaviours serious enough to violate the basic rights of others or to exceed age-appropriate societal norms or rules. Three-month prevalence rates of DSM-IV-TR defined CD averaged 1.2 % in girls and 4.2 % in boys in a large longitudinal cohort study from the US [12] and the prevalence of CD was 2.7 % in a representative sample of 5- to 16-year olds in Great Britain [13]. CD is a matter of significant public health and societal concern because of strong associations with adverse scholastic and work performance, disrupted peer and family relationships, excessive risk-taking behaviours which often result in personal injury, loss of property, alcohol, nicotine and substance abuse, accident proneness and development of later antisocial personality disorder [14, 15]. In addition, CD has also been associated with multiple service use [13].

The treatment of CD rests primarily on reducing the number and severity of aggressive behaviours perpetrated by patients with CD. Pharmacotherapy is usually not the first line of treatment but is considered in those subjects who have failed to respond to other interventions. Potential non-pharmacological treatments include psychosocial/behavioural interventions such as cognitive therapy or social learning family interventions, e.g. parent management training [16–18]. The pharmacological options for the treatment of CD can be categorised into psychostimulants (mostly in cases with attention deficit hyperactivity disorder—ADHD), antipsychotics, mood stabilisers and various other less-used treatments such as α_2_ receptor agonists and β-blockers [19].

## Risperidone efficacy in children and adolescents with CD

The US Food and Drug Administration (FDA) approved risperidone in children and adolescents in the following indications: (1) treatment of schizophrenia in adolescents, ages 13–17; (2) short-term treatment of bipolar mania associated with manic or mixed episodes of bipolar I disorder in children and adolescents aged 10–17; (3) irritability associated with autistic disorder in children and adolescents aged 5–17 (Risperdal prescribing information http://www.accessdata.fda.gov/scripts/cder/drugsatfda/index.cfm.

In Europe, risperidone has been approved for the short-term treatment (6 weeks) of aggression in CD in children from the age of 5 and adolescents with sub-average intellectual functioning or mental retardation (details in: http://www.ema.europa.eu/docs/en_GB/document_library/Referrals_document/Risperdal_30/WC500007979.pdf).

The efficacy of risperidone in the disruptive behaviour disorders [a broad category including CD, oppositional defiant disorder (ODD), and also disruptive behaviour disorder—not otherwise specified] and aggression has mostly been studied in sub-average IQ children and adolescents. A summary of randomised controlled trials of risperidone in children and adolescents with these disorders and aged 5–18 is shown in Table 1. A meta-analysis of two of the above studies using the conduct problem sub-score of the Nisonger Child Behaviour Rating Form showed a mean difference of −8.61 (95 % CI −11.49, −5.74) judged likely to be clinically significant [20].

**Table 1:** Summary of short-term randomised controlled trials of risperidone in disruptive behaviour disorders in children and adolescents 5 years and above

Reference	Sample (age) IQ	Duration	Dose (mg/day)	Primary outcome Mean change (SD) placebo/risperidone (*p*) Effect size
Findling 2000	*N* = 20 (5–12)	10 weeks	0.75–1.5	RAAPP
	IQ > 70			−0.16 (0.54)/−1.65 (0.40) (*p* = 0.03)
Buitelaar 2001	*N* = 38 (12–18)	6 weeks	2.9	CGI-S
	IQ 60–90			(*p* < 0.01)
Van Bellinghen 2001	*N* = 13 (6–18)	4 weeks	1.2	ABC irritability (not prespecified)
	IQ 45–85			0.1 (9.4)/−10.8 (6.05) (*p* < 0.05)
Aman 2002	*N* = 118 (5–12)	6 weeks	1.16	NCBRF-CP
	IQ 34–84			−6.2 (11.2)/−15.2 (10.6) (*p* < 0.001)
				−0.82
Snyder 2002	*N* = 110 (5–12)	6 weeks	0.98	NCBRF-CP
	IQ 36–84			−6.8/−15.8 (*p* < 0.01)
				−0.73
Reyes 2006	*N* = 335 (5–17)	6 months	<50 kg 0.81	Time to symptom recurrence^a^
	216 with IQ > 84		>50 kg 1.22	Symptom recurrence in 25 %: 37 versus 119 days (*p* < 0.001)
	119 with IQ < 84			
Armenteros 2007	*N* = 25 (7–12)	28 days	1.08	CAS-P and CAS-T ≥ 30 % change
	IQ > or = 75			CAS-P total: 77 %/100 % (*p* < 0.5)
	All with ADHD			CAS-T total: 54 versus 27 % (NS)
				No differences in mean CAS scores.

*RAAPP* rating of aggression against people and/or property scale, *CGI-S* Clinical Global Impression-Severity, *NCBRF-CP* Nisonger Child Behaviour Rating Form conduct problem subscale, *CAS-P* Children’s Aggression Scale-Parent, *CAS-T* Children’s Aggression Scale-Teacher, *NS* non significant

^a^Deterioration of two points or more on the Clinical Global Impression-Severity or seven points of more on the conduct problems subscale

A pooled analysis of nine RCTs of atypical antipsychotics that measured aggressive behaviour examined a total of 875 subjects (mean age = 9.2 years) [21]. On average, study length was 45.7 days and the mean dose of risperidone was 0.04 mg/kg/day. Effect sizes were generally large (mean ES = 0.9) and increased with study duration. Risperidone was associated with greater effect sizes than other medication classes such as mood stabilisers, stimulants, antidepressants and α2 agonists.

To determine the long-term effectiveness of risperidone for severe disruptive behaviours in children, 504 patients were enrolled into in a multisite, 1-year, open-label study of patients aged 5–14 years with disruptive behaviours and sub-average intelligence [22]. Seventy-three percent of those enrolled completed the study and the mean dose of risperidone was 1.6 mg/day. Scores on the Nisonger Child Behaviour Rating Form Conduct Problem Scale improved significantly as early as week 1, and improvement was maintained throughout the trial (*p* < 0.001 at each time point).

A small subsample of patients (48 patients), who had previously completed the 1-year open-label study of risperidone, was followed-up for an additional year of treatment [23]. The efficacy benefits from the original study were maintained over the course of this extension study.

The above data support the short-term efficacy of risperidone in children with sub-average IQ, but evidence is limited in adolescents and youngsters with normal intelligence. Long-term efficacy of risperidone has only been documented in children with sub-average IQ or mixed samples. Moreover, the potential impact of ADHD on treatment response is unknown; ADHD status has not been specified in the majority of the above studies. The same is true for psychosocial interventions, also likely to affect treatment outcome.

## Risperidone safety in children and adolescents with CD

The paediatric population may be more vulnerable to metabolic, endocrine, and extrapyramidal adverse effects of SGAs than adults [24]. The Canadian Alliance for Monitoring Effectiveness and Safety of Antipsychotics in Children (CAMESA) guidelines group analysed 57 articles on the use of risperidone in children and adolescents [25]. Main adverse effects found when comparing patients receiving risperidone with those on placebo were the following: (1) higher mean weight gain with a mean difference of 2.09 kg in randomised controlled trials (RCTs) lasting 6 months, (2) higher odds of extrapyramidal side effects (OR 3.55, *p* < 0.00001), (3) elevated prolactin levels at endpoint in RCTs 12 weeks or less (with a mean difference of 899.99 pmol).

Open-label studies showed continuous weight gain and BMI increase; treatment of children during 45 weeks has lead to a total mean weight gain of 7.2 kg with risperidone, 16.2 kg with olanzapine and 9.5 kg with clozapine [26]. A large, prospective, open-label, non-randomised study in youths aged 4–19, naive to antipsychotic medications, and treated for almost 11 weeks, showed a weight gain equal or above 7 % in 64.4 % of patients with risperidone, in 84.4 % receiving olanzapine, in 58.4 % with aripiprazole and in 0 % comparison subjects [27]. In a recent review, Number Needed to Harm (NNH) for weight gain with risperidone was estimated between 7 and 8 in children and adolescents [28].

In a combined dataset from five clinical trials in 5- to 15-year-old children treated with risperidone, serum prolactin showed a raise with a peak observed after 2 months of treatment, followed by a gradual return to normal levels after 5 months [29], but other studies have shown persistence of high levels of prolactin in subjects treated with risperidone 1 year or more [30]. In a pubertal sample of males aged 10–20, receiving long-term risperidone (mean duration 52 months), hyperprolactinemia was present in 47 versus 2 % in those without treatment and 14 versus 0 % reported sexual dysfunction [31]. Clinical consequences of the increase of prolactin levels are inconsistent across studies and effects may vary with dose, treatment duration and the pubertal status of patients [32].

The analysis of a series of placebo-controlled studies showed that risperidone induced extrapyramidal effects in a variable percentage of patients between 8 and 26 %. NNH for tremor, dyskinesia or other extrapyramidal symptoms varied from 6 to 33 % for risperidone according to dose and duration [28]. A recent meta-analysis reported higher odds of extrapyramidal symptoms in children treated with risperidone (OR 3.55; 95 % CI 2.04−5.48) and in those treated with aripiprazole (OR 3.70; 95 % CI 2.37–5.77) compared with placebo. Sedation is another commonly reported adverse effect of risperidone, mostly occurring at the start of treatment or at after a dose increase. Reported NNH for sedation, somnolence or drowsiness varies between 2 and 5 [28].

Currently, there are insufficient data available about the long-term safety of risperidone in children and adolescents. The majority of safety studies have been limited to 1 year, while treatment in clinical practice is typically for longer periods. This is particularly important as the long-term use of risperidone is not without health risks and is associated with several potential adverse reactions, of which weight gain and associated metabolic disruption are perhaps the most worrisome.

## Aims and objectives of PERS

The PERS clinical trials will focus on the investigation of short-term efficacy, safety/tolerability and maintenance effects of risperidone in the treatment of children and adolescents with CD and *normal* intelligence, since in this patient population risperidone has not systematically been studied. Another focus of PERS is the long-term safety that will be assessed in an observational pharmacovigilance study, regardless of indication and patient characteristics. As a first step, we developed a clinical trial strategy and submitted a number of specific questions to the European Medicine Agency (EMA) on the potential design of the studies, the choice of the pivotal measures and their potential impact for granting a Paediatric Use Marketing Authorisation. These recommendations as well as those from the EMA on the Paediatric Investigational Plan (PIP) application were incorporated into the current trial protocols. Close collaboration has been established with a manufacturer of risperidone and a contract research organisation that will be able to implement and monitor clinical psychopharmacology studies in this age group on a European level. PERS will also address a number of secondary exploratory questions that will be discussed below.

## Clinical trials in PERS

PERS consist of three related clinical studies, a short-term efficacy and safety study, a study on maintenance effects, and an open-label observational study. The three study designs including details regarding clinical and/or laboratory parameters are outlined in detail as part of the Schedule of Events (SOE; Annex 1).

### Short-term efficacy of risperidone in CD: conduct disorder in children and adolescents (CONCA)

CONCA is a multicentre, randomised, double-blind, parallel, placebo-controlled trial. This RCT will examine whether risperidone, given orally in a dose of 0.25–3.0 mg/day for 12 weeks, is superior to placebo in reducing disruptive behavioural symptoms of children and adolescents with CD without mental retardation. CONCA comprises three study periods and aims to enrol 264 patients (50 % children 5- to 11-years old and 50 % adolescents 12–17.5 years). The sample size calculations were based on an assumption of an effect size of 0.4 and 90 % power with a 1:1 randomization scheme to risperidone or placebo. The clinical diagnosis of CD according to DSM-IV-TR will be confirmed by a structured interview (Kiddie-SADS, conduct disorder module [33] and a score ≥27 on the ODD/CD composite score of the Nisonger CBRF will be required at Visits 2 or 3 to meet criteria of sufficiently severe CD). Children and adolescents with ADHD and those on stable stimulant medication, with no planned changes during the trial, will not be excluded (see supplementary material in Annex 1 for details of inclusion and exclusion criteria).

Study Period I is a 2-week screening and washout period; during this period, patients will be screened for study eligibility. Randomization to risperidone or placebo will occur at Visit 3. Subjects will randomly be assigned to risperidone or placebo in a 1:1 ratio and randomisation will not be stratified. Study Period II is a 12-week acute treatment period (Visits 3–9). In order to safeguard patients in the study, patients can be either in outpatient or inpatient settings. For patients in the risperidone treatment group, medication will be given in the evening and dosing will begin at either 0.25 (<50 kg body weight) or 0.5 (≥50 kg), mg/day (tablets), depending on the patient’s weight. Doses will be increased by 0.25 mg/day or 0.5 mg/day increments each week to maximum doses that vary by patient weight (<50 kg or ≥50 kg), based on the investigator’s assessment of efficacy and tolerability/safety. Study Period III is a 1-week double-blind period, a progressive withdrawal from study medication (Visits 9–10).

Efficacy is measured by the last observation carried forward (LOCF) mean change from baseline to endpoint on the pivotal scale, the NCBRF–Typical IQ Version [34]. The primary measure is the ODD/CD Disruptive Behaviour Composite Total score using investigator ratings based on all available information. The decision to use an investigator-rated NCBRF in the PERS studies is due to regulatory reasons (i.e. because in registration trials there is a strong preference for a primary outcome based on professional/clinical assessment rather than an assessment by parents, patients or teachers).

Secondary objectives of the study are to examine whether treatment with risperidone is associated with lower symptoms on the Clinical Global Impression: Improvement and Severity scales (CGI-I and CGI-S [35], the Modified Overt Aggression Scale (M-OAS [36, 37]), and with better functioning on the Child-Global Assessment Scale (C-GAS [38]), the CHIP-CE Parent Report Form [39] and the Positive Social subscale of the Nisonger CBRF Typical IQ version [34]. We will also assess the effect of risperidone on concurrent ADHD symptoms using the ADHD-Rating Scale [40] and examine whether ADHD and its treatment influence the risperidone effect on disruptive behaviours.

We plan to further assess the effect of risperidone compared to placebo on cognitive functioning (e.g. due to possible sedative effects) using attentional and set-shifting tests from the Amsterdam Neuropsychological Test battery (ANT) [41]. Safety and tolerability will be assessed extensively and include spontaneously reported and investigator-rated treatment-emergent adverse events based on information from both patient and parents. Measures comprise potential extrapyramidal symptoms (Barnes Akathisia Scale [42], Simpson-Angus Scale [43], Abnormal Involuntary Movement Scale (AIMS) [35]); changes in ECG, vital signs, body temperature, and laboratory analyses; changes in growth, assessed using height, weight, and body mass index (BMI); and changes in suicidal ideation and suicidal behaviour, as assessed by the investigator-rated Columbia Suicide Severity rating Scale (C-SSRS) [44].

### Maintenance effects in CD: discontinuation for conduct disorder in children and adolescents (DIS-CONCA)

DIS-CONCA is a multicentre, randomised, double-blind, parallel, placebo-controlled discontinuation (relapse prevention) trial in children and adolescents, aged 6–17.5, with CD and without mental retardation. The study incorporates a screening phase, an open-label period of treatment with risperidone followed by randomization to continued risperidone or placebo substitution over a 12-week period. This allows for an evaluation of rates of relapse following response, and it is an accepted method for determining the value of differing lengths of continuation treatment following an initial response.

DIS-CONCA will examine whether risperidone given orally in a dose of 0.25–3.0 mg/day is superior to placebo in preventing relapse of symptoms of CD, as assessed through a 12-week, double-blind discontinuation trial. Inclusion criteria are similar to those for CONCA, and the pivotal scale is as in CONCA the NCBRF–Typical IQ Version-ODD/CD disruptive behaviour Composite Total score [34] using investigator ratings based on all available information. Since compliance is often poor in CD children and adolescents, significant non-compliance [missing more than two consecutive days of study medication (full doses), or failure to take at least 70 % of prescribed doses of study medication (full doses) during two or more visit intervals] will be discussed with patient and parent and the patient will be discontinued only when, in the opinion of the investigator, the patient is deemed unlikely to become compliant and data obtained from the patient judged unreliable.

Clinical response is defined as >25 % reduction from baseline score on the NCBRF-TIQ D-Total subscale at endpoint and a score of 1 or 2 (“much” or very much” improved) on the CGI-Improvement scale. The primary efficacy measure will be comparison of the number of days from randomization to relapse for each treatment group using the Kaplan–Meier product limit estimator. Relapse is defined as a deterioration of >2 points on the CGI-Severity scale and a 25 % increase in the score on the Nisonger pivotal scale, compared to start of Study Period II (average of Visits 7 and 8), for at least two consecutive visits, 15 days apart.

Secondary objectives of DIS-CONCA are to establish the long-term efficacy of treatment with risperidone on secondary measures as the CGI-S, C-GAS, and Overt Aggression Scale (OAS) and several measures of functioning as the Child-Global Assessment Scale (C-GAS;[57]), the CHIP-CE Parent Report Form [6] and the ‘Positive Social’ subscale of the Nisonger CBRF Typical IQ version [1]. Further, we will assess the long-term effects of risperidone on cognitive functioning and on ADHD symptoms, and collect extensive data on safety and tolerability.

Participants will undergo four study periods. After screening (Study Period I), Study Period II will be a 16-week titration and open-label treatment period (7 visits). This acute treatment period was chosen to increase the chance to reflect the change based on medication treatment. All patients who complete Study Period II and maintain clinical response are eligible to participate in Study Period III. Two consecutive positive evaluations that meet the criteria for response are required to enter into the randomised discontinuation phase. Study Period III will be a 12-week double-blind, randomised, placebo-controlled discontinuation period (8 visits). At the start of Study Period III, patients will be randomised to continued risperidone or placebo (1:1 ratio). In the placebo-discontinuation group, risperidone gradual discontinuation will be started during the first 4 weeks of Study Period III, but the specific timing of starting the discontinuation will be unknown to patients, their families and the investigators. Patients who meet relapse criteria during Study Period III will be considered completers and may be discontinued and given the opportunity of entering the observational prospective study. Therefore, no interim analysis to stop the study prematurely has been planned in the discontinuation phase. After 24 weeks, medication will be discontinued in 2 weeks in all the non-relapsing patients (Study Period IV); patients will have their final clinical assessment after further 2 weeks, with the opportunity of entering, if appropriate, the observational prospective study.

Based on power calculations on previous studies with risperidone in CD with mild mental retardation, 150 (50 % children 6–11, 50 % adolescents 12–17.5) patients should be enrolled in Study Period II. A sample size of 150 using a 1:1 randomization over risperidone and placebo will have 80 % power to detect an increase in relapse rate from 33.3 % in the risperidone group to 66.6 % in the placebo group, using a proportional hazards analysis with a censoring of data following a uniform distribution between the 6th and the 12th week (simulations, functionspower, libraryHmisc, R software 2.14). Considering that 25 % of patients enrolled in Study Period II will not meet inclusion criteria to continue in Study Period III, 200 patients should be enrolled in Study Period II.

### Observational pharmacovigilance study

There is a lack of knowledge about which factors make some children and adolescents more vulnerable than others to the short-term and long-term adverse effects of risperidone [8, 45–47]. Dose may be one obvious mediator, but many available studies did not obtain risperidone plasma concentrations. Some recent evidence has suggested that genetic factors that may also influence the occurrence of metabolic or endocrine side effects such as weight gain and hyperprolactinemia, but much remains to be investigated in this area [48, 49]. Finally, little is known about the degree of reversibility of these adverse reactions upon treatment discontinuation. The pharmacovigilance study within PERS is aimed at filling in the safety knowledge gap, through conducting an observational open-label study in which 600 children and adolescents aged between 5 and 16 years who have been prescribed risperidone will be followed over a treatment period of up to 2 years and comparing them to 250 controls. If there has been previous use of risperidone, children can also enter the study as long as there has been no use of risperidone for the past 6 months. The study will be able to detect a difference in the rates of clinically significant weight gain over the first year of usage, defined as an increase in BMI standardised score of 0.5 or greater. We plan to follow 600 patients under medication and this will allow detection of an increase from 3 per 100 to 8 per 100 with 90 % power. The study has further 95 % power to detect rare adverse events, i.e. with an incidence rate as low as 0.5 per cent. All participating patients will be regular referrals to child and adolescent psychiatry centres. There will be no restrictions for the reasons of prescribing risperidone, both labelled and unlabelled indications will be included. Also, any possible concomitant medication will be allowed. The pharmacovigilance study will be fully embedded within regular clinical practice and will solely collect data from patients’ medical records, without imposing any impact on choice and duration of treatment. Participating clinical centres have therefore agreed on systematic safety monitoring, in accordance with international recommendations [25].

Main objectives of the PERS pharmacovigilance study are to follow the course of BMI, waist circumference and biological parameters and to systematically collect the frequency, nature, and course of subjectively reported possible side effects. Subjects will be enrolled at the start of risperidone treatment. The PERS pharmacovigilance study includes clinical and/or laboratory parameters prior to and after 1, 2, 3, 6, 12, 18, and 24 months of treatment will be collected. Subjectively reported possible side effects, including those related to suicidality and sexuality will be collected. Blood laboratory parameters include glycosylated haemoglobin A1, liver enzymes, fasting lipids (triglycerides, total cholesterol, HDL cholesterol, LDL cholesterol); fasting blood glucose; fasting insulin; and prolactin (see supplementary information for details about Schedule of Events).

We will also investigate moderating and/or mediating factors of the short- and long-term adverse events and changes in weight and waist circumference. Specifically, we will examine the influence of (1) medication factors (average daily dose; cumulative dosage of risperidone; pre-treatment history of medication; use of concomitant medication; duration of treatment; discontinuation versus continued use); (2) patient characteristics (age; Tanner stage; initial BMI/waist circumference; socioeconomic status; clinical efficacy; clinical indication), and (3) lifestyle factors including dietary factors, levels of physical activity, and illicit drug use.

A hierarchical linear regression model will be used to compare the BMI standardised scores over time with adjustment for the correlations between assessments within the same individual. Similar models will be used to identify patterns that may be associated with drug usage for the secondary outcomes.

### Secondary and exploratory studies on moderating factors

In addition to examining main treatment effects, it is important to also examine the impact of potential moderating factors of efficacy and safety. Moderators are characteristics of subjects before treatment that have an impact on efficacy or tolerability.

An important topic is the subtyping of aggressive and antisocial behaviour. Among children and adolescents who develop severe patterns of aggressive and antisocial behaviour, there are certain subgroups that may be subject to distinct causal processes that result in their problem behaviour. Specifically, callous-unemotional (CU) traits (e.g. lack of guilt, absence of empathy, callous use of others) are relatively stable and are associated with more severe conduct problems, delinquency, or aggression. Children and adolescents with CD with and without CU traits also differ in their emotional, cognitive, and personality characteristics [50]. Another important distinction has been made between reactive forms of aggression (e.g. in response to perceived provocation or treat) and instrumental aggression (e.g. premeditated aggression for some gain or reward) [51]. Post hoc, we will examine whether risperidone differentially affects CD with and without CU traits, and instrumental versus affective aggression in both CONCA and DIS-CONCA trials. We will also examine the role of co-occuring ADHD, because children with CD and with concurrent ADHD have an earlier onset of CD and a worse outcome compared to children with CD without ADHD [52]. Moderators of the short- and long-term adverse events occurring during risperidone treatment will also be analysed in the observational pharmacovigilance study.

## Ethical considerations

PERS has formed an ethics working group which aims: (1) to guarantee that ethical standards defined by international guidelines and national legislations are adhered to and, (2) to conduct empirical research on ethical issues arising around PERS. Ethical problems related to research with children, a so-called vulnerable population, have been poorly studied. Legislative ethical guidelines [e.g. International Conference on Harmonisation (ICH) of technical requirements for registration of pharmaceuticals for human use and Good Clinical Practice, (GCP), EU Regulation 1901/2006] implemented in the US and Europe aim to safeguard children in clinical research and also to facilitate research with this group. Study-specific ethical issues for PERS include (1) the placebo design, i.e. has the placebo group been withheld from receiving an effective treatment?, (2) how can a valuable informed consent/assent of minors be guaranteed? and (3) the guarantee of only minimal harm and burden in the study (are additional blood-taking and study examinations acceptable to the children?).

## Training

Consistent and simultaneous study performance across several European countries with different nationalities and languages require standard operation procedures (SOPs). The study personnel are required to comply with GCP guidelines, be equipped with clear instructions and feel safe concerning all relevant procedures. As a key condition for successful study conduct, a common standard of rating has to be ensured. This requires a clear structured and harmonised training programme for both junior and senior study personnel across the various countries and study sites. To facilitate inter-site contact, to introduce the study procedures and to train all involved researchers and professional groups (physicians, psychologists, nurses, CROs), we organised training workshops over a 2-year period. These had a consistent content to ensure that the study protocols and procedures were reliably taught to study personnel.

Training sessions aimed to reach consistent inter-rater reliability across sites in the application of the Nisonger Child Behaviour Rating Form, relevant to decisions about study participation (a D score of at least 27 is mandatory) and used as the primary outcome measure. Therefore, three case vignettes were developed and two of them were sent to the participating centres prior to the workshop, with the request to rate them and to compare the ratings with the suggested “expert” rating by the Ulm group. During the workshop, the third videotape was used to assess agreement on each item and the resulting subscales. Results for the 18 raters showed that mean value of the D score was 35.4 (SD = 4.75; range 27–45). Eight raters (44.4 %) were in the range of 34–37 and four had a score of exactly 35, which is also the “expert score”. As the designated proceedings concerning adverse events (AE) are corresponding to ICH GCP guidelines, no extra checking in addition to the one by the monitor was planned.

The rating standards have been documented in booklets, and are also available as downloadable pdf documents on the restricted area of the project website. Annual repetition of training workshops is scheduled both to maintain the awareness of the procedures among previously trained staff and train new incoming personnel.

## Discussion

As a consequence of the significant burden on the patient, family and immediate environment and the strong associations with adverse scholastic and work performance, disrupted peer and family relationships, excessive risk-taking behaviours and addictive behaviours, CD is a matter of significant public health and societal concern [53, 54].

Although behavioural parent training programmes have been shown to have some effects in children and other psychological treatment approaches may be promising for adolescents with CD [17], there is an urgent need for further evidence-based effective behavioural and pharmacological treatments for CD in children and adolescents. More effective treatments will have the potential to not only prevent the negative personal consequence of CD and increase both personal productivity and contribution to society, but could also reduce the burden of CD on the family and society. Whilst risperidone is often prescribed off-label for CD, there is a clear need to provide evidence for its use in youth with CD and normal IQ. PERS studies will also provide support to professional bodies developing guidelines for diagnosis and treatment of CD. Furthermore, it is anticipated that PERS will address several scientific questions about the moderating and/or mediating factors of the short-term efficacy and maintenance of clinical response, and the short-term and long-term safety of risperidone in children and adolescents with CD. In turn, better understanding of these moderating and mediating factors will help clinicians make evidence-based decisions in the consultation room.

### Strengths and challenges in PERS

PERS is an academic project financed by the European Union, involving expert centres in child and adolescent psychopharmacology all over Europe that collaborates through the European College of Neuropsychopharmacology (ECNP) child and adolescent network. While the paediatric regulations implemented in US and Europe have been an important step for the development of research in child and adolescent neuropsychopharmacology, involvement of professional networks and associations is crucial for the implementation of research and training in academic settings. Recent examples of this support are the creation of the ECNP school of child and adolescent neuropsychopharmacology and specialised workshops and expert meetings during ECNP congresses [2]. Therefore, the goals of this collaborative project extend beyond the broadening of approved indications of risperidone and also include a series of issues that are important for treatment optimization. The latter include the identification of risk factors for metabolic side effects, the effects of risperidone on cognitive performance and long-term safety of risperidone in CD and in other chronic conditions such as autism and schizophrenia.

We also expect that PERS will have impact on research in paediatric psychopharmacology by establishing a collaborative network of expert centres, based on the ECNP child and adolescent neuropsychopharmacology network (www.ecnp.eu), and that the dissemination of results will lead to improve standards of care through the development of assessments and guidelines. Although CD requires multidisciplinary and social interventions, providing safe and effective medications for the treatment of CD is expected to lower the burden of this condition on individuals, family and society.

PERS, however, is not without challenges. The regulatory framework is tight and complex, and the regulatory requirements and associated timelines are difficult to reconcile with the straightforward time planning of milestones and deliverables in a typical EU grant. Specifically, the need to obtain approval from all national regulatory bodies and ethics commissions was source of unexpected delays and called for numerous revisions of the study protocol. This point makes it also difficult to consider any changes or amendments once the protocol has been approved, because any further delays will impact the feasibility of patient recruitment and available budget. Furthermore, budget has to be allocated for a supporting infrastructure that involves a Contract Research Organization (CRO), a clinical trial database, a central lab and ECGs, and central management of drug supply and logistics. Future non-commercial clinical trials with EU funding will need to take all these into account. It should also be noted by the European Commission that the budget to run clinical trial with sufficient quality should be larger than it is at present.

Last but not least, we have to face the difficulties in recruiting a sufficient number of patients and their families for the clinical studies. CD populations are relatively understudied in clinical studies and trials compared to, for example, ADHD populations. Many children with CD are not willing to follow a treatment and in a high percentage of cases have significantly disrupted family and social backgrounds; therefore, getting informed consent and ensuring compliance is likely to be a significant issue. A common observation is that CD is relatively underdiagnosed in mental health settings, due to fear for social stigma, and instead a less-stigmatised diagnosis of ODD is conferred. PERS will be instrumental in putting CD higher at the research agenda in Europe and activating clinicians and mental health professionals in recognising, diagnosing and treating CD.

### Electronic supplementary material

Below is the link to the electronic supplementary material.

Supplementary material 1 (DOC 640 kb)

## PERS Consortium:

All the above authors and the following:

Loes Vinkenvleugel (RUNMC; l.vinkenvleugel@karakter.com).

Rosalia Lavado Autric (SERMES/PSN; rosalia_lavado@sermes.org).

Gordon Urquhart (Wockhardt Ltd, UK; gordon.urquhart@wockhardt.co.uk).

Sarah Curran (KCL; sarah.curran@kcl.ac.uk).

Ruth Berg (CIMH; Ruth.Berg@zi-mannheim.de).

Tobias Banaschewski (CIMH; tobias.banaschewski@zi-mannheim.de).

Alexander Haege (CIMH; Alexander.Haege@zi-mannheim.de).

David Cohen (AP-HP GH Pitié-Salpêtrière; david.cohen@psl.aphp.fr).

Marie Raffin (AP-HP GH Pitié-Salpêtrière; marie.raffin@psl.aphp.fr).

Laura Pina-Camacho (SERMAS/HGUGM; lpina@iisgm.com).

Ana Calvo (SERMAS/HGUGM; acalvo@iisgm.com).

Goretti Moron (SERMAS/HGUGM; gmoron@iisgm.com).

Simon Schlanser (UULM; simon.schlanser@uniklinik-ulm.de).

Sonia Aslan (UULM; sonia.aslan@uniklinik-ulm.de).

Ferdinand Keller (UULM; ferdinand.keller@uniklinik-ulm.de).

Jacinta Tan (UULM; jmcl2007@gmail.com).

Cora Drent (UMCG; c.drent@accare.nl).

Paola Brovedani (IRCCS Fondazione Stella Maris; p.brovedani@inpe.unipi.it).

Jan Steyaert (KU Leuven; jean.steyaert@uzleuven.be).

Astrid Morer (Hospital Clínic of Barcelona; amorer@clinic.ub.es)

Shaun Treweek (UNIVDUN; streweek@mac.com).

Sara Bahadori (AP-HP Hôpital R.Debré; sabahadori@yahoo.fr).

Hugo Peyre (AP-HP Hôpital R.Debré; peyrehugo@yahoo.fr).

Alexandre Hubert (AP-HP Hôpital R.Debré; axhubert@gmail.com).

Carla Balia (UNICA; balia.carla@gmail.com).

Immaculada Baeza (Hospital Clínic of Barcelona; ibaeza@clinic.ub.es).

Luisa Lazaro (Hospital Clínic of Barcelona; llazaro@clinic.ub.es).

Fiona Hogarth (Tayside Clinical Trials Unit, Ninewells Hospital & Medical School, Dundee; f.j.hogarth@dundee.ac.uk).

Susan MacFarlane, Children’s Research Unit, Ninewells Hospital, Dundee; susanmacfarlane@nhs.net).

Ekta Patel; Division of Neuroscience, Ninewells Hospital, Dundee; e.patel@dundee.ac.uk).

Sarah Seth; Division of Neuroscience, Ninewells Hospital, Dundee, sarahseth@nhs.net).

Ewa Nowotny; (Dept of Child Psychiatry, King’s College London, London; ewa.nowotny@kcl.ac.uk).

Regina Sala; (Dept of Child Psychiatry, King’s College London, London; regina.sala_cassola@kcl.ac.uk).
