# Supplementary material for: Paediatric European Risperidone Studies (PERS): context, rationale, objectives, strategy, and challenges
Source: Eur Child Adolesc Psychiatry. 2013 Dec 15;23(12):1149–60. doi: 10.1007/s00787-013-0498-3 (PMC4246122; doi:10.1007/s00787-013-0498-3)
Supplement: Supplementary file 1 — Supplementary material 1 (DOC 640 kb) [file 787_2013_498_MOESM1_ESM.doc]

**PERS-CONCA– supplementary information**

**Study Population**

This study will include male and female inpatients or outpatients with disruptive behaviours associated with Conduct Disorder (CD). Patients are eligible for enrolment in the study if they meet all of the inclusion criteria and none of the exclusion criteria described below.

**Inclusion Criteria**

Patients are eligible to be included in the study only if they meet **all** of the inclusion criteria below.

[1] Male or female patients, aged 5;0 - < 17;9 years at Visit 1***.***

[2] Patients must have an IQ of > 85 (based on,> 4 subtests, 2 verbal plus 2 performance tests) from the Wechsler IQ Scales, e.g. WISC; WAIS; assessed within < 2 y. before or at study entry), e.g.: vocabulary, similarities, block design, and matrix reasoning (cf. Crawford et al., 2010). Age- and country-specific adaptations may be used (detailed instructions may be provided in a respective extra document).

[3] Patients must meet diagnostic criteria for DSM-IV TR Conduct Disorder(s), as confirmed by the Kiddie-SADS, Conduct Disorder Module: 312.8x. (Kaufman et al., 1996), at Visit 2 **or** Visit 3.

[4] Patients must score ≥ 27 on the Nisonger CBR Form, ODD/CD Disruptive Behavior Composite (D-Total) at Visit 2 **or** Visit 3.

[5] Patients must score ≥4 (“moderately ill”) on the CGI-S rating scale at Visits 2 **and** 3.

[6] If a female of  child-bearing potential, patients must test negative for pregnancy at the time of enrollment based on a serum pregnancy test and agree to use a reliable method of birth control. (Adequate contra- ception includes: oral contraceptives, intrauterine devices; double barrier method (diaphragm or condom plus spermicide), Norplant™ or Depot Provera™).

[7] Patients must have a body weight of at least 20 kg at study entry.

[8] Patients must be able to swallow study drug.

[9] Patients must have venous access sufficient to allow blood sampling and are compliant with blood draws as per protocol.

[10] Subjects’ parents/legal guardians must provide and sign informed consent documents; patients must provide informed consent, and sign consent or assent documents if capable, according to the legal requirements in the very country.

[11] A reliable person (primary caregiver, parent) must be available to ensure compliance with study procedures throughout the course of the study.

[12] Parents and patients must have a level of education, understanding and command of language suitable to adequately communicate with investigator and study coordinator.

[13] Patients meeting criteria for comorbid ADHD (as to the clinical judgment of the investigator) will not be excluded from study participation.

## 4.2. Exclusion Criteria

A patient will be excluded from the study if he or she meets any exclusion criteria described below, according to the assessment of the investigator.

[14] Is immediate family of investigator site personnel directly affiliated with this study. Immediate family is defined as a spouse, parent, child, or sibling, whether biological or legally adopted.

[15] Has been treated with a drug within 14 days before Visit 1 that has not received regulatory approval for any indication at the time of study entry.

[16] Has participated in any investigational drug trial within six months prior to baseline (visit 3).

[17] Has previously completed or withdrawn from this study or any other study investigating risperidone or has previously been identified as being a nonresponder or intolerant of risperidone.

[18] In the clinical judgment of the investigator, has a current (within 6 months of the start of the study) or lifetime DSM-IV-TR diagnosis of schizophrenia-related disorders, schizophrenia, bipolar disorder, major depressive disorder, or current substance dependence disorder (given the nature of the study population substance misuse or abuse is not exlusionary), pervasive developmental disorder (autistic disorder or Asperger disorder).

[19] In the clinical judgment of the investigator, currently meets criteria for a primary psychiatric disorder, e.g., Anxiety Disorder, Depressive Disorder, Tic Disorder or Tourette’s Syndrome (comorbid ADHD is permitted, cf. Incl. criteria section)

[20] Starts any psychotropic medication, including health-food supplements that the investigator feels could have central nervous system activity (for example, St. John’s Wort, melatonin), during the course of the study, or is taking any other excluded concomitant medication(s) at/beyond Visit 2 (specified in Section 5.7). (An ongoing long-term medication, e.g., to treat a comorbid disorder such as ADHD, is permitted as long as compound and dose are not changed throughout the course of the study.)

[21] Has any acute or unstable medical condition **such as renal or hepatic dysfunction**, physiological condition, clinically significant laboratory, or ECG results that, in the opinion of the investigator, would compromise participation in the study.

[22] Has a known or suspected seizure disorder.

[23] Has a history of neuroleptic malignant syndrome (NMS) or of tardive dyskinesia.

[24] Has a history of severe allergies to medications, in particular hypersensitivity to neuroleptics, or of multiple adverse drug reactions.

[25] Is pregnant or nursing.

**CONCA –Flow Chart**

**
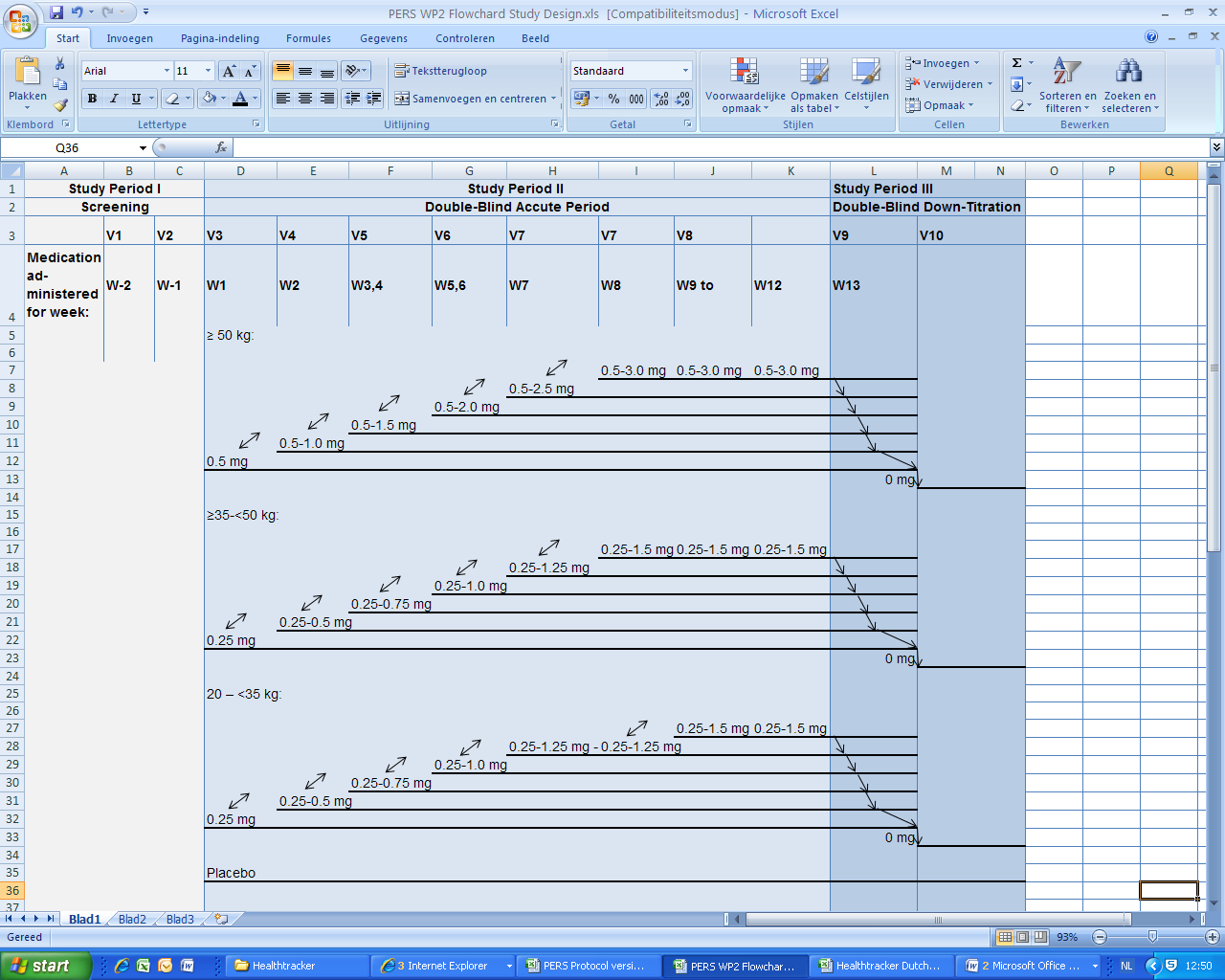
**

Study Schedule of Events (SOE)

| Study Period | SP I (Screen/W.) | | | SP II (Double-Blind Treatment) | | | | | | | | | | | | SP III Down-titration | | | |  |  |
| --- | --- | --- | --- | --- | --- | --- | --- | --- | --- | --- | --- | --- | --- | --- | --- | --- | --- | --- | --- | --- | --- |
| **Visit Number:** | **1** | **2** | | **3** | | **4** | | **5** | | **6** | | **7** | | **8** | | **9** | | **10** | | **Unsched.**  **visits** | Early Disc. |
| **Week (end of):** | **-2** | **-1** | | **0** | | **1** | | **2** | | **4** | | **6** | | **8** | | **12** | | **13** | |  |  |
| **Suggested Time to Next Visit (days):** | **7** | **5-9** | | **7** | | **7** | | **14** | | **14** | | **14** | | **28** | | **7** | |  | |  | **na** |
| **Allowable Time to next Visit (days):** | **5-9** |  | | **5-9** | | **5-9** | | **10-18** | | **10-18** | | **10-18** | | **24-32** | | **5-9** | |  | |  | **na** |
| **Clinic Assessments** |  | | | | | | | | | | | | | | | | | | | |  |
| **Demographics and baseline characteristics** |  | |  | |  | |  | |  | |  | |  | |  | |  | |  |  |  |
|  |  | |  | |  | |  | |  | |  | |  | |  | |  | |  |  |  |
| Informed consent/assent | x | |  | |  | |  | |  | |  | |  | |  | |  | |  |  |  |
| k-SADS: (CD) (vis. 2 **or** 3) |  | | x | | x | |  | |  | |  | |  | |  | |  | |  |  |  |
| Inclusion/exclusion criteria | x | | x | | x | |  | |  | |  | |  | |  | |  | |  |  |  |
| Demographics | x | |  | |  | |  | |  | |  | |  | |  | |  | |  |  |  |
| Physical examination | x | |  | |  | |  | |  | |  | |  | |  | |  | |  |  |  |
| Medical history | x | |  | |  | |  | |  | |  | |  | |  | |  | |  |  |  |
| Psychiatric assessment | x | |  | |  | |  | |  | |  | |  | |  | |  | |  |  |  |
| Presenting/preexisting diseases (conditions) and medications | x | | x | | x | |  | |  | |  | |  | |  | |  | |  |  |  |
| Discontinuation of prohibited medicines | x | |  | |  | |  | |  | |  | |  | |  | |  | |  |  |  |
| Confirm washout/prohibited medicines |  | | x | | x | |  | |  | |  | |  | |  | |  | |  |  |  |
|  |  | |  | |  | |  | |  | |  | |  | |  | |  | |  |  |  |
| **Safety Measures** |  | |  | |  | |  | |  | |  | |  | |  | |  | |  |  |  |
|  |  | |  | |  | |  | |  | |  | |  | |  | |  | |  |  |  |
| Vital signs (HR, BP) | x | |  | | x | | x | | x | | x | | x | | x | | x | | x | x | x |
| Height | x | |  | |  | |  | |  | |  | |  | |  | | x | |  |  | x |

**Study Schedule of Events (SOE)**

| Study Period | SP I (Screen/W.) | | SP II (Double-Blind Treatment) | | | | | | SP III Down-titration | |  |  |
| --- | --- | --- | --- | --- | --- | --- | --- | --- | --- | --- | --- | --- |
| **Visit Number:** | **1** | **2** | **3** | **4** | **5** | **6** | **7** | **8** | **9** | **10** | **Unsched.**  **visits** | Early Disc. |
| **Week (end of):** | **-2** | **-1** | **(0)** | **1** | **2** | **4** | **6** | **8** | **12** | **13** |  |
|  |  |  |  |  |  |  |  |  |  |  |  |  |
| Weight | x |  | x |  |  | x |  |  | x |  |  | x |
| ECG | x |  | x |  |  | x |  | x | x |  | x | x |
| Tanner staging |  |  | x |  |  |  |  |  | x |  |  | x |
| AE monitoring |  | x | x | x | x | x | x | x | x | x | x | x |
| PAERS |  |  | x | x | x | x | x | x | x | x | x | x |
| C-SSRS/  Self-Harm suppl,/follow-up^2^ | x | x | x | x | x | x |  |  | x | x | x | x |
| EPS (SAS, BAS, AIMS) |  |  | x | x | x | x |  | x | x |  | x | x |
| ANT test battery |  |  | x |  |  | x |  |  | x |  |  | x |
| Assessment reconsent/assent (cf. 9.1, p. 48);  - CBCA and  - RKQ instruments  - motivational questions |  |  | x |  |  |  |  |  | x |  |  | x |
|  |  |  |  |  |  |  |  |  |  |  |  |  |
| **Laboratory Tests**^3^ |  |  |  |  |  |  |  |  |  |  | 5 |  |
|  |  |  |  |  |  |  |  |  |  |  |  |  |
| Drug of abuse screen (Urine DS) | x |  |  |  |  |  |  |  |  |  | x |  |
| Pregnancy test (females^4^); urine |  |  | x |  |  | x |  |  | x |  | x | x |
| Pregnancy test (females^4^); serum | x |  |  |  |  |  |  |  |  |  |  |  |
| Urinanalysis | x |  | x |  |  | x |  |  | x |  |  | x |

**Study Schedule of Events (SOE)**

| Study Period | SP I (Screen/W.) | | SP II (Double-Blind Treatment) | | | | | | SP III Down-titration | |  |  |
| --- | --- | --- | --- | --- | --- | --- | --- | --- | --- | --- | --- | --- |
| **Visit Number:** | **1** | **2** | **3** | **4** | **5** | **6** | **7** | **8** | **9** | **10** | **Unsched.**  **visits** | Early Disc. |
| **Week (end of):** | **-2** | **-1** | **(0)** | **1** | **2** | **4** | **6** | **8** | **12** | **13** |  |  |
|  |  |  |  |  |  |  |  |  |  |  |  |  |
| Clinical chemistry^5^ | x |  | x |  |  | x |  |  | x |  |  | x |
| Electrolytes | x |  | x |  |  | x |  |  | x |  |  | x |
| Glucose (fasting^6^) | x |  | x |  |  | x |  |  | x |  |  | x |
| Lipids (fasting^6^) | x |  | x |  |  | x |  |  | x |  |  | x |
| HgbA1c |  |  | x |  |  |  |  |  | x |  |  | x |
| Prolactin | x |  | x |  |  | x |  |  | x |  |  | x |
| Hematology | x |  | x |  |  | x |  |  | x |  |  | x |
| Risperidone level (if possible) |  |  |  |  |  |  |  | x or | x |  |  | x |
|  |  |  |  |  |  |  |  |  |  |  |  |  |
| **Study Procedures** |  |  |  |  |  |  |  |  |  |  |  |  |
|  |  |  |  |  |  |  |  |  |  |  |  |  |
| Randomization |  |  | x |  |  |  |  |  |  |  |  |  |
| Study drug dispensed |  |  | x | x | x | x | x | x | x |  |  |  |
| Dose titration regimen |  |  | x | x | x | x | x | x | x |  |  |  |
| Dose fixed |  |  |  |  |  |  |  | x |  |  |  |  |
| Study drug compliance/adherence ; MARS |  |  |  | x | x | x | x | x | x | x |  | x |
| Concomitant medications | x | x | x | x | x | x | x | x | x | x | x | x |

**Study Schedule of Events (SOE)**

| Study Period | SP I (Screen/W.) | | SP II (Double-Blind Treatment) | | | | | | SP III Down-titration | |  |  |
| --- | --- | --- | --- | --- | --- | --- | --- | --- | --- | --- | --- | --- |
| **Visit Number:** | **1** | **2** | **3** | **4** | **5** | **6** | **7** | **8** | **9** | **10** | **Unsched. Visits** | Early Disc. |
| **Week (end of):** | **-2** | **-1** | **(0)** | **1** | **2** | **4** | **6** | **8** | **12** | **13** |  |
|  |  |  |  |  |  |  |  |  |  |  |  |  |
| **Efficacy Measures** |  |  |  |  |  |  |  |  |  |  |  |  |
|  |  |  |  |  |  |  |  |  |  |  |  |  |
| Nisonger CBRF-TIQ |  | x | x | x | x | x | x | x | x | x |  | x |
| CGI-S; CGI-**I (from vis 4)** | x | x | x | x | x | x | x | x | x | x |  | x |
| M-OAS |  |  | x |  |  | x |  |  | x | x |  | x |
| C-GAS |  |  | x |  |  | x |  |  | x |  |  | x |
| CHIP-CE |  |  | x |  |  | x |  |  | x |  |  | x |
|  |  |  |  |  |  |  |  |  |  |  |  |  |
| CBCL/6-18 |  |  | x |  |  |  |  |  |  |  |  |  |
| ADHD-RS |  |  | x |  |  | x |  |  | x |  |  | x |

**^1^** assessed within < 2 y. before or at study entry

**^2^** if indicated

**^3^**of child-bearing potential only; additionally at the discretion of the investigator

**^4^**Labs, ECG, etc. at Unscheduled Visits (due to AEs) according to the discretion of the investigator

**^5^**patients with significant increase after baseline (vis. 3) in AST, ALT, totoal bilirubin, or AP from the upper limit of the lab

reference range will have additional lab testing and/or consultation

**^6^**to be collected in a fasting state at visits 3 and 9 (and early disc.); fasting defined as >/= 8 h with water as the only oral consumption

**DIS CONCA- Flow chart**


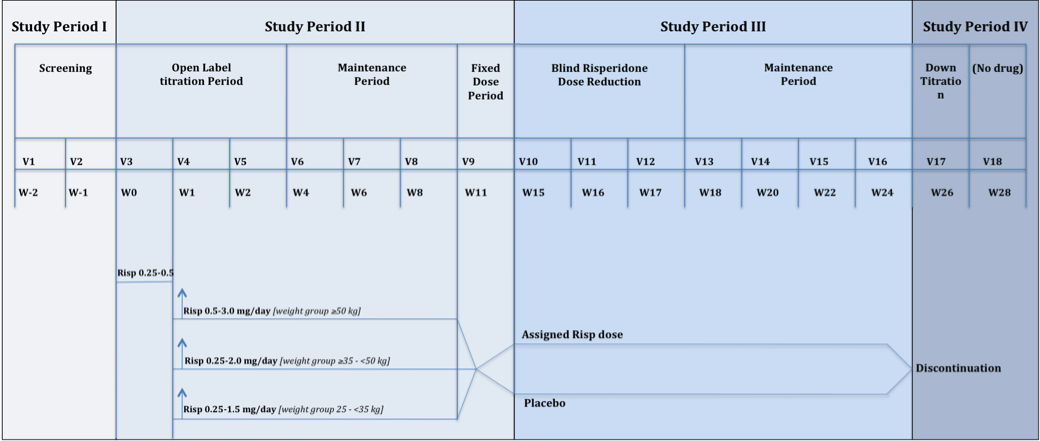


DIS_CONCA Risperidone Dosing Schedule during Study Period II, in mg/day, by Baseline Body Weight

| **Baseline Body Weight (kg)** | **V3**  **(Wk 0)** | **V4**  **(Wk 1)** | **V5**  **(Wks 2-3)** | **V6**  **(Wks 4-5)** | **V7**  **(Wk 6-7)** | **V8**  **(Wk 8-10)** | **V9**  **(Wk 11-14)** |
| --- | --- | --- | --- | --- | --- | --- | --- |
|  |  |  |  |  |  |  |  |
| **≥50** | **0.5** | **0.5 – 1.0** | **0.5 – 1.5** | **0.5 – 2.0** | **0.5 – 2.5** | **0.5 – 3.0** | **0.5 – 3.0** |
|  |  |  |  |  |  |  |  |
|  |  |  |  |  |  |  |  |
| **≥35–<50** | **0.25** | **0.25 – 0.5** | **0.25 – 1.0** | **0.5 – 1.5** | **0.5 – 2.0** | **0.5 – 2.0** | **0.5 – 2.0** |
|  |  |  |  |  |  |  |  |
|  |  |  |  |  |  |  |  |
| **25 -< 35** | **0.25** | **0.25 – 0.5** | **0.25 – 0.75** | **0.5 – 1.0** | **0.5 – 1.0** | **0.5 – 1.0** | **0.5– 1.0** |
|  |  |  |  |  |  |  |  |
| **No. of tablets** | **1** | **1 - 2** | **1 - 3** | **1 - 3** | **1 - 3** | **1 - 3** | **1 - 3** |
|  |  |  |  |  |  |  |  |

**DIS-CONCA Schedule of Events (SOE)**

|  | **SP I** | | **SP II** | | | | | | | **SP III** | | | | | | | **SP IV** | |  |
| --- | --- | --- | --- | --- | --- | --- | --- | --- | --- | --- | --- | --- | --- | --- | --- | --- | --- | --- | --- |
|  | **Screening** | | **Open-Label Treatment Period** | | | | | | | **Double-Blind Discontinuation Period** | | | | | | | **Down- Titration** | **No drug** | **Early Disc.** |
|  |  |  | **Up-Titration** | | | **Maintenance Dose** | | | **Fixed Dose** | **Blind Risperidone**  **dose reduction** | | | **Maintenance Dose** | | | |  |  |  |
| **Visit** | **1** | **2^*^** | **3** | **4** | **5** | **6** | **7** | **8** | **9** | **10** | **11** | **12** | **13** | **14** | **15** | **16** | **17** | **18** |  |
| **Assessment week** | **-2** | **-1** | **0** | **1** | **2** | **4** | **6** | **8** | **11** | **15** | **16** | **17** | **18** | **20** | **22** | **24** | **26** | **28** |  |
| **Suggested time to next visit (days)** | 7 | 7 | 7 | 7 | 14 | 14 | 14 | 21 | 28 | 7 | 7 | 7 | 14 | 14 | 14 | 14 | 14 | End of study |  |
| Informed consent/assent | X |  |  |  |  |  |  |  |  |  |  |  |  |  |  |  |  |  |  |
| Inclusion/exclusion criteria | X |  |  |  |  |  |  |  |  |  |  |  |  |  |  |  |  |  |  |
| Demographics | X |  |  |  |  |  |  |  |  |  |  |  |  |  |  |  |  |  |  |
| Medical and Mediaction History | X |  |  |  |  |  |  |  |  |  |  |  |  |  |  |  |  |  |  |
| Psychiatric Assessment | X |  |  |  |  |  |  |  |  |  |  |  |  |  |  |  |  |  |  |
| K-SADS | X |  |  |  |  |  |  |  |  |  |  |  |  |  |  |  |  |  |  |
| Presenting/preexisting diseases (conditions) and medications | X |  |  |  |  |  |  |  |  |  |  |  |  |  |  |  |  |  |  |
| Discontinuation of prohibited medicines | X |  |  |  |  |  |  |  |  |  |  |  |  |  |  |  |  |  |  |
| Confirm washout/prohibited medicines | X |  |  |  |  |  |  |  |  |  |  |  |  |  |  |  |  |  |  |
| IQ (Wechsler, 4 subtests;cf. Incl. criteria for details) | X |  |  |  |  |  |  |  |  |  |  |  |  |  |  |  |  |  |  |
| Physical Examination | X |  |  |  |  | X |  |  | X | X |  |  |  |  |  |  |  | X | X |
| Height | X |  |  | X | X | X | X | X | X | X | X | X | X | X | X | X | X | X | X |
| Weight/BMI | X |  |  | X | X | X | X | X | X | X | X | X | X | X | X | X | X | X | X |
| Body Temperature | X |  |  | X | X | X | X | X | X | X | X | X | X | X | X | X | X | X | X |
| Vital signs | X |  |  | X | X | X | X | X | X | X | X | X | X | X | X | X | X | X | X |
| ECG | X |  |  |  | X |  |  |  | X |  |  |  |  | X |  |  |  | X | X |

|  | **SP I** | | **SP II** | | | | | | | **SP III** | | | | | | | **SP IV** | |  |
| --- | --- | --- | --- | --- | --- | --- | --- | --- | --- | --- | --- | --- | --- | --- | --- | --- | --- | --- | --- |
|  | **Screening** | | **Open-Label Treatment Period** | | | | | | | **Double-Blind Discontinuation Period** | | | | | | | **Down- Titration** | **No drug** | **Early Disc.** |
|  |  |  | **Up-Titration** | | | **Maintenance Dose** | | | **Fixed Dose** | **Blind Risperidone**  **dose reduction** | | | **Maintenance Dose** | | | |  |  |  |
| **Visit** | **1** | **2^*^** | **3** | **4** | **5** | **6** | **7** | **8** | **9** | **10** | **11** | **12** | **13** | **14** | **15** | **16** | **17** | **18** |  |
| **Assessment week** | **-2** | **-1** | **0** | **1** | **2** | **4** | **6** | **8** | **11** | **15** | **16** | **17** | **18** | **20** | **22** | **24** | **26** | **28** |  |
| **Suggested time to next visit (days)** | 7 | 7 | 7 | 7 | 14 | 14 | 14 | 21 | 28 | 7 | 7 | 7 | 14 | 14 | 14 | 14 | 14 | End of study |  |
| Clinical lab test | X |  |  |  | X |  |  |  | X |  |  |  |  |  | X |  |  | X | X |
| Drug of abuse screen (Urine DS) | X |  |  |  | X |  |  |  | X |  |  |  |  |  | X |  |  | X | X |
| Serum pregnancy test (females*); urine | X |  |  |  | X |  |  |  | X |  |  |  |  |  | X |  |  | X | X |
| Urinanalysis | X |  |  |  | X |  |  |  | X |  |  |  |  |  | X |  |  | X | X |
| Risperidone plasma level |  |  |  |  |  |  |  |  | X |  |  |  |  |  |  |  |  |  |  |
| Investigator dose assessment |  |  | X | X | X | X | X | X | X | X | X | X | X | X | X | X | X |  |  |
| Investigator dose adjustment |  |  |  | X | X | X | X | X | X | X | X | X |  |  |  |  |  |  |  |
| Dose titration regimen |  |  | X | X | X |  |  |  |  | X | X | X |  |  |  |  | X | X |  |
| Dose fixed |  |  |  |  |  |  |  |  | X |  |  |  | X | X | X | X |  |  |  |
| Randomization |  |  |  |  |  |  |  |  |  | X |  |  |  |  |  |  |  |  |  |
| Study drug distributed |  |  | X | X | X | X | X | X | X | X | X | X | X | X | X | X | X |  |  |
| Study drug returned |  |  |  | X | X | X | X | X | X | X | X | X | X | X | X | X | X | X | X |
| Compliance assessed |  |  |  | X | X | X | X | X | X | X | X | X | X | X | X | X | X | X | X |
| Concomitant medication | X |  | X | X | X | X | X | X | X | X | X | X | X | X | X | X | X | X | X |
|  |  |  |  |  |  |  |  |  |  |  |  |  |  |  |  |  |  |  |  |

|  | **SP I** | | **SP II** | | | | | | | **SP III** | | | | | | | **SP IV** | |  |
| --- | --- | --- | --- | --- | --- | --- | --- | --- | --- | --- | --- | --- | --- | --- | --- | --- | --- | --- | --- |
|  | **Screening** | | **Open-Label Treatment Period** | | | | | | | **Double-Blind Discontinuation Period** | | | | | | | **Down- Titration** | **No drug** | **Early Disc.** |
|  |  |  | **Up-Titration** | | | **Maintenance Dose** | | | **Fixed Dose** | **Blind Risperidone**  **dose reduction** | | | **Maintenance Dose** | | | |  |  |  |
| **Visit** | **1** | **2^*^** | **3** | **4** | **5** | **6** | **7** | **8** | **9** | **10** | **11** | **12** | **13** | **14** | **15** | **16** | **17** | **18** |  |
| **Assessment week** | **-2** | **-1** | **0** | **1** | **2** | **4** | **6** | **8** | **11** | **15** | **16** | **17** | **18** | **20** | **22** | **24** | **26** | **28** |  |
| **Suggested time to next visit (days)** | 7 | 7 | 7 | 7 | 14 | 14 | 14 | 21 | 28 | 7 | 7 | 7 | 14 | 14 | 14 | 14 | 14 | End of study |  |
| Prior adverse events and TEAs | X |  | X | X | X | X | X | X | X | X | X | X | X | X | X | X | X | X | X |
| PAERS |  |  |  | X | X | X | X | X | X | X | X | X | X | X | X | X | X | X | X |
| BAS- SAS- AIMS |  |  |  | X | X | X | X | X | X | X | X | X | X | X | X | X | X | X | X |
| C-SSRS | X |  |  | X | X | X | X | X | X | X | X | X | X | X | x | x | x | X | X |
| Nisonger CBRF | X |  | X | X | X | X | X | X | X | X | X | X | X | X | X | X | X | X | X |
| CGI-S | X |  | X | X | X | X | X | X | X | X | X | X | X | X | X | X | X | X | X |
| CGI-I |  |  | X | X | X | X | X | X | X | X | X | X | X | X | X | X | X | X | X |
| C-GAS | X |  | X | X | X | X | X | X | X | X | X | X | X | X | X | X | X | X | X |
| M-OAS | X |  |  |  | X | X |  |  | X | X | X | X |  | X |  |  | X | X | X |
| CBCL (only problem behaviour) | X |  |  |  |  |  |  |  |  |  |  |  |  |  |  |  |  |  |  |
| CHIP-CE: PRF-76 | X |  |  |  | X |  |  |  | X |  |  |  |  |  |  |  | X |  | X |
| ADHD-RS | X |  |  |  | X | X |  |  | X | X | X | X | X | X | X | X | X | X | X |
| ANT (4 subtests) | X |  |  |  |  |  |  |  | X |  |  |  |  |  |  |  | X |  | X |
| Satisfaction Q. |  |  |  |  |  |  |  |  |  |  |  |  |  |  |  |  |  | X | X |
| CBCA/ RKQ computerized | X |  |  |  |  |  |  |  |  |  |  |  |  |  |  |  |  | X | X |
| Tanner staging | X |  |  |  |  |  |  |  |  |  |  |  |  |  |  |  |  | X | X |

* Screening procedures may be performed on two different days (1-10 days apart: Visit 1 and Visit 2).

**PERS Observational study – supplementary information : Schedule of Events (SOE)**

| **Measurement** | **Baseline** | **Month 1** | **Month 2** | **Month 3** | **Month 6** | **Month 12** | **Month 18** | **Month 24*** |
| --- | --- | --- | --- | --- | --- | --- | --- | --- |
| *Visit time* |  | *3-5* | *7-9* | *10-16* | *23-29* | *49-55* | *75-81* | *101-107* |
| DSM-IV TR | X |  |  |  |  |  |  |  |
| Review current and lifetime use of medication | X |  |  |  |  |  |  |  |
| Family History | X |  |  |  |  |  |  |  |
| Socio-economic status | X |  |  |  |  |  |  |  |
| CHIP-CE: PRF 76 | X |  |  | X | X | X |  | X |
| IWQOL-KID | X |  |  | X | X | X |  | X |
| Biochemistry: | X |  |  | X | X | X |  | X |
| Physical Examination) | X | X | X | X | X | X | X | X |
| UKU | X | X | X | X | X | X | X | X |
| AIMS | X | X | X | X | X | X | X | X |
| PAQ-C or PAQ-A | X | X | X | X | X | X | X | X |
| HABITS | X | X | X | X | X | X | X | X |
| Tanner stage | X | X | X | X | X | X | X | X |
| Reviiew Illicit drug use | X | X | X | X | X | X | X | X |
| CGI-S | X | X | X | X | X | X | X | X |
| CGI-I |  | X | X | X | X | X | X | X |
| Changes in medication |  | X | X | X | X | X | X | X |
